# Between-sex variability of resting state functional brain networks in amyotrophic lateral sclerosis (ALS)

**DOI:** 10.1007/s00702-021-02413-0

**Published:** 2021-09-01

**Authors:** Francesca Trojsi, Federica Di Nardo, Giuseppina Caiazzo, Mattia Siciliano, Giulia D’Alvano, Carla Passaniti, Antonio Russo, Simona Bonavita, Mario Cirillo, Fabrizio Esposito, Gioacchino Tedeschi

**Affiliations:** Department of Advanced Medical and Surgical Sciences, MRI Research Center SUN-FISM, Università degli Studi della Campania “Luigi Vanvitelli”, 80138 Naples, Italy

**Keywords:** Amyotrophic lateral sclerosis, Resting-state functional MRI, Brain networks, Functional connectivity, Sex

## Abstract

The organization of brain functional connectivity (FC) has been shown to differ between sexes. Amyotrophic lateral sclerosis (ALS) is characterized by sexual dimorphism, showing sex-specific trends in site of onset, phenotypes, and prognosis. Here, we explored resting state (RS) FC differences within major large-scale functional networks between women and men in a sample of ALS patients, in comparison to healthy controls (HCs). A group-level independent component analysis (ICA) was performed on RS-fMRI time-series enabling spatial and spectral analyses of large-scale RS FC networks in 45 patients with ALS (20 F; 25 M) and 31 HCs (15 F; 16 M) with a focus on sex-related differences. A whole-brain voxel-based morphometry (VBM) was also performed to highlight atrophy differences. Between-sex comparisons showed: decreased FC in the right middle frontal gyrus and in the precuneus within the default mode network (DMN), in affected men compared to affected women; decreased FC in the right post-central gyrus (sensorimotor network), in the right inferior parietal gyrus (right fronto-parietal network) and increased FC in the anterior cingulate cortex and right insula (salience network), in both affected and non-affected men compared to women. When comparing affected men to affected women, VBM analysis revealed atrophy in men in the right lateral occipital cortex. Our results suggest that in ALS sex-related trends of brain functional and structural changes are more heavily represented in DMN and in the occipital cortex, suggesting that sex is an additional dimension of functional and structural heterogeneity in ALS.

## Introduction

Different performances between sexes on various cognitive skills, including executive control (Gaillard et al. 2021a), language (Wegesin 1998), spatial thinking (Zancada-Menendez et al. 2016; Gur and Gur 2017), emotional processing (Whittle et al. 2011) and social cognition (Gur and Gur 2017), have been explained in the last decades on the base of between-sex dissimilarities in brain function (Gaillard et al. 2021b; Whittle et al. 2011; Stevens and Hamann 2012; Volf et al. 2010; Filippi et al. 2013; Gur and Gur 2017). This evidence has supported the hypothesis that males and females engage different strategies depending on task demands related to different neural networks and may indicate adaptive diversity and complementarity between the sexes (Gur and Gur 2017). Furthermore, this aspect may represent a critical step toward a better understanding of sex-related pathological processes underlying most neurologic and psychiatric disorders characterized by sexual dimorphism (Lawrence et al. 2020; De Micco et al. 2019; Bakeberg et al. 2021). This, in turn, would help develop in the future personalized treatments for different neurological disorders tailored for male and female populations, respectively.

An appealing unbiased strategy to assess activity differences between sexes is the evaluation of resting-state (RS) functional connectivity (FC). In recent years, FC fMRI approaches found several brain regions whose spontaneous low-frequency fluctuations (< 0.1 Hz) of the blood oxygen level-dependent (BOLD) signal registered during RS correlate with each other. Those regions are believed to be functionally connected (Biswal et al. 2010; Greicius et al. 2003; van den Heuvel and Hulshoff Pol 2010). Based on their functional and/or anatomical overlap with well-known large-scale functional networks, distinct resting-state functional networks (RSNs) have been identified (Damoiseaux et al. 2006; Seeley et al. 2007), representing the brain’s function beyond explicit tasks and, consequently, the intrinsic functional architecture of the human brain (Smith et al. 2009). Using independent component analysis (ICA), several studies have demonstrated the occurrence of high temporal correlations between spatially distinct but functionally related brain regions, resembling specific neuroanatomical networks, which characterize the RSNs (Damoiseaux et al. 2006; Tedeschi and Esposito 2012). Furthermore, the spectral composition of RS-fMRI signals, as quantified by the fractional amplitude of low-frequency fluctuations (fALFF) (Zou et al. 2008) across four canonical frequency sub-bands (slow-5, 0.01–0.027 Hz; slow-4, 0.027–0.073 Hz; slow-3, 0.073–0.198 Hz; slow-2, 0.198–0.25 Hz) has been also considered to further characterize the BOLD time-courses within the RSNs under pathological (neurodegenerative) conditions (Esposito et al. 2013).

Recent studies explored the influence of sex on the RSNs, revealing that men showed stronger connectivity in parieto-temporal regions, and within cognitive and sensory networks, while women displayed stronger connectivity in fronto-temporo-cerebellar regions, and within attention and memory-related networks (Filippi et al. 2013). Moreover, RS-fMRI and graph theoretical approaches, used to investigate the hemisphere- and gender-related differences in brain functional networks in 86 young, healthy, right-handed adults, showed that males tended to be more locally efficient in the right hemispheric networks, but females tended to be more locally efficient in the left hemispheric networks (Tian et al. 2011).

Among neurodegenerative disorders characterized by sexual dimorphism (Pinares-Garcia et al. 2018), amyotrophic lateral sclerosis (ALS), a devastating motor neuron disease causing the progressive impairment of motor function (speech, swallowing, limb, respiration), has been shown to be strongly influenced by sex, together with age and genetic variations, in phenotypic manifestation (i.e. onset, phenotype and early progression of motor and cognitive symptoms) (Chiò et al. 2017, 2020; Rooney et al., 2017a, b; Trojsi et al. 2019). Sex has been reported as an independent factor involved in the development of ALS, with the higher risk of exhibiting the disease in men (Chiò et al. 2017), especially in case of flail limbs and respiratory phenotypes, with a trend toward a higher frequency in older age (Chiò et al. 2020). Moreover, the role of male sex in clinical presentation and prognosis of ALS patients carrying repeat expansions in chromosome 9 open reading frame 72 (*C9orf72*) has been also explored (Rooney et al. 2017a; Trojsi et al. 2019). Nevertheless, while there is consolidated clinical and neurobiological evidence in favor of sexual dimorphism in ALS, from the neuroimaging point of view, only one MRI study investigated sex differences in the structural patterns of cortical and subcortical pathology in ALS (Bede et al. 2014). In particular, Bede et al. (2014) revealed major between-sex differences in diffusion tensor imaging (DTI) and cortical thickness measures in fronto-temporal and cerebellar regions.

On this background, in order to start shedding some light on the still unclear functional MRI brain correlates of sex differences in ALS, we performed a RS-fMRI analysis with ICA, quantifying component-level fALFF, in a cohort of 45 patients with classical ALS compared to 30 healthy subjects, stratifying both cohorts for sex. In addition, we performed a whole-brain voxel-based morphometry (VBM) analysis to assess gray matter (GM) volume changes associated to sex differences within the same cohorts. We expected to find gender-related RS-fMRI and VBM patterns of brain damage useful to better characterize the differences in brain function and structure between sexes in ALS patients.

## Methods

### Case selection

Forty-five right-handed patients (25 males, 20 females; mean age 57.6 ± 9.5), with definite, clinical or laboratory-supported probable ALS, according to El-Escorial revised criteria (Brooks et al. 2000), were consecutively recruited at the First Division of Neurology of the University of Campania “Luigi Vanvitelli” (Naples, Italy) from November 2018 to February 2020. Patients were required to meet the following criteria: classical, bulbar, flail limbs or upper motor neuron (UMN) dominant phenotypes (Chiò et al. 2011); symptom onset not earlier than 18 months from enrollment to principally include ALS patients with mild or moderate disability, probably in early stages of disease, representative of the populations which are more commonly recruited in clinical trials (van Eijk et al. 2020a, b), and able to lie supine during MRI scan; age of onset of 40 years or older.

The clinical assessment included: assessment of disability status, measuring the ALSFRS-R total score (0–48, with lower total reflecting higher disability) and subscores (i.e., bulbar, fine-motor, gross-motor and respiratory subscores) (Cedarbaum et al. 1999) and the upper motor neuron (UMN) score, index of pyramidal dysfunction through the evaluation of the number of pathologic reflexes elicited from 15 body sites (Turner et al. 2004); assessment of global cognitive functioning, administering the Italian version of Edinburgh Cognitive and Behavioural ALS Screen (ECAS) (Poletti et al. 2016; Siciliano et al. 2017). Disease duration was calculated from symptom onset to scan date in months, and the rate of progression determined by: (48 minus current ALSFRS-R)/disease duration. Disease stage was assessed according to King’s clinical staging system (Roche et al. 2012). Genetic analysis was performed in all patients, exploring *C9orf72* repeat expansion and mutations of *SOD1*, *TARDBP* and *FUS/TLS*. No mutations of these genes were reported.

Thirty right-handed healthy control subjects (HCs) (15 males, 15 females; mean age 55.2 ± 9.1) were enrolled by “word of mouth” and among caregivers’ friends. They were age-, sex- and education-matched with the enrolled ALS patients and unrelated to them. Moreover, they had no comorbid neurological, psychiatric or medical conditions. They underwent MMSE and their scores were ≥ 27 points.

Exclusion criteria for all subjects were: medical illnesses or substance abuse that could interfere with cognitive functioning; any (other) major systemic, psychiatric, or neurological diseases; other causes of brain damage, including lacunae and extensive cerebrovascular disorders at MRI; a vital capacity lower than 70% of the predicted value (as a cut-off for respiratory dysfunction, which may hinder to lie supine during the scan).

The research was conducted according to the principles expressed in the Declaration of Helsinki. Ethics approval was obtained from the Ethics Committee of the University of Campania “Luigi Vanvitelli”. Patient/HC or family written informed consent was obtained from each participant.

### Statistical analysis: between-groups comparisons of clinical and neuropsychological data

All data were tested for normality, and values between − 1 and + 1 for asymmetry were considered acceptable (Hays et al. 1993). Males and females belonging to ALS and HC groups were compared by one-way analyses of variance (one-way ANOVAs). All comparisons were corrected according to the Bonferroni procedure. The analyses were performed using the Statistical Package for Social Science (SPSS) version 21, with *p* value < 0.05 considered statistically significant.

### MRI analysis

#### Magnetic resonance imaging

MR images were acquired on a 3 T scanner equipped with an 8-channel parallel head coil (General Electric Healthcare, Milwaukee, Wisconsin). The imaging protocol included: three-dimensional T1-weighted sagittal images (gradient-echo sequence Inversion Recovery prepared Fast Spoiled Gradient Recalled-echo, time repetition = 6.988 ms, TI = 1100 ms, TE = 3.9 ms, flip angle = 10, voxel size = 1 × 1 × 1.2 mm^3^; acquisition time = about 10 min) (Cavedo et al. 2014); RS-fMRI was performed with a gradient-echo echo-planar imaging (GRE-EPI) sequence generating of 240 T2*-weighted volumes of 29 axial slices (time repetition = 1508 ms, echo time = 32 ms, FA = 90°, voxel size = 4 × 4 × 4 mm3, matrix = 64 × 64, field of view = 256 mm, slice thickness = 4 mm, interslice gap = 0 mm; total acquisition time =  ~ 6 min); T2-fluid attenuation inversion recovery to exclude severe cerebrovascular disease according to standard clinical neuroradiological criteria on visual inspection by three experienced radiologists. During the functional scan, subjects were asked to simply stay motionless, awake, and relax and to keep their eyes closed. No visual or auditory stimuli were presented at any time during functional scanning. The total duration of each scan was about 38 min.

#### RS-fMRI data preparation and preprocessing

Standard functional image data preparation and preprocessing, statistical analysis, and visualization were performed with the software BrainVoyager QX (Brain Innovation BV, Maastricht, The Netherlands). Data preprocessing included the correction for slice scan timing acquisition, a three-dimensional rigid-body motion correction based on a 6-parameter rigid-body alignment to correct for minor head movements, and the application of a temporal highpass filter with cut-off set to 3 cycles per time-course. Translational motion parameters were verified to be always less than 1 functional voxel for all included participants. The mean frame-wise displacement (FD) (i.e., a surrogate metric of head motion accounting for intra-voxel residual motion effects) was also estimated from the translational and rotational parameters and a typical cut-off of 0.5 mm was applied (Power et al. 2014). We further verified that there were no statistically significant differences in the mean FD when carrying the same group comparisons. Structural and functional data were coregistered and spatially normalized to the Talairach standard space using a 12-parameter affine transformation. During this procedure, the functional images were resampled to an isometric 3-mm grid covering the entire Talairach box. Finally, the resulting image time series were spatially smoothed with a 6-mm full-width half-maximum isotropic Gaussian kernel.

#### Resting state network (RSN) functional connectivity analysis

To extract RSN maps, single-subject and group-level independent component analyses (ICA) were carried out on the preprocessed functional time series using two plug-in extensions of BrainVoyager QX (Goebel et al. 2006), respectively, implementing the fastICA algorithm (Hyvärinen et. 2001) and the self-organizing group ICA algorithm (Esposito et al. 2005). Furthermore, an ICASSO step was added for the extraction of ICA components. ICASSO (Himberg et al. 2004) is a validated procedure to ensure the maximal algorithmic and statistical stability of ICA components of neuroimaging time-series. ICASSO entails with running the FastICA algorithm many times (in our study we set the number of repetitions to 20) with different initial values (algorithmic reliability) and with differently bootstrapped data sets (statistical reliability). This procedure allows extracting the most stable ICA decomposition via hierarchical clustering of all generated ICA components at the selected dimensionality. The stability of ICASSO-derived decompositions can be expressed by two quantitative indices: the R-index, expressing the average cluster compactness, and the B-index, expressing the balance among clusters. The R-index measures the mean inner distance of the clusters relative to the distance from their nearest neighbor, thereby lower values (e.g., *R* < 1) indicate more stable decompositions, whereas a B-index closer to 100% indicates a higher balance in the number of components contributing to each cluster, i.e., a lower chance that clusters had been formed with either too many or too few components.

For each single subject, 40 independent components were extracted (corresponding to 1/6th of the number of time points) (Grecius et al. 2007) and scaled to spatial *z* scores (i.e., the number of standard deviations of their whole-brain spatial distribution). To generate group components and allow for group-level inferences in each RSN, all individual component maps from all subjects were “clustered” in the subject space according to the mutual similarities of their whole-brain distributions using the self-organizing group ICA algorithm. Thereby, all 40 individual independent components were uniquely assigned to 1 out of 40 “clusters” of independent components. Once the components belonging to a cluster were selected, the corresponding maps were averaged and the resulting group map was taken as the representative FC pattern of the cluster. The 40 single-group average maps were visually inspected to recognize the spatial patterns associated with the main RSNs (Smith et al. 2009). For this purpose, single-group 1-sample *t* tests were used to analyze the whole-brain distribution of the components in each group separately and the resulting t maps were thresholded at *p* = 0.05 (Bonferroni corrected over the entire brain) after regressing out age, gender and (for patients) disease duration from the series of individual maps at each voxel. An inclusive mask was finally created from the healthy control group maps and used to define the search volume for within-network 2-group comparisons. These comparisons were performed by fitting a one-way analysis of variance (ANOVA) model that included one between-subject factor with four levels: ALS affected males (ALS-M), ALS affected females (ALS-F), HC males (HC-M) and HC females (HC-F) and then calculating post hoc t contrasts for obtaining between-group t maps. To correct the resulting t maps for multiple comparisons, regional effects within the search volume were only considered significant for compact clusters emerging from the joint application of a voxel- and a cluster-level threshold. The cluster-level threshold was estimated non-parametrically with a randomization approach: we calculated the FWHM from each RSN t map for the HC group and then, starting from an initial (uncorrected) threshold of *p* = 0.001 or *p* = 0.005 applied to all voxels, a minimum cluster size was calculated that protected against false-positive clusters at 5% after 1000 Monte Carlo simulations (Forman et al. 1995).

Component-specific spectral power data were obtained using the fast Fourier transform (FFT) function in Matlab (The Mathworks, USA) applied to each component time-courses. In this way, subject- and component-specific spectral power information was made available. For each group, group- and component-specific spectral power information was obtained from the average of subject-specific spectral power data across homologue components. From subject- and component-specific spectral power, the fALFF of each network was characterized as follows: for each network component time-course power spectrum, the relative contribution of four separate bands were calculated (network fALFF). Following Zuo et al. (2010a), four canonical bands were considered: slow-5 (0.01–0.027 Hz), slow-4 (0.027–0.073 Hz), slow-3 (0.073–0.198 Hz) and slow-2 (0.198–0.25 Hz). Finally, individual fALFF scores for both the patient and control groups were used for between-group comparisons.

#### Regional atrophy measurements: voxel-based morphometry (VBM)

We performed a VBM analysis using SPM12 software package (http://www.fil.ion.ucl.ac.uk/spm/) with default parameters incorporating the DARTEL toolbox to obtain a high-dimensional normalization protocol (Ashburner 2007). Data processing was carried out according to a previously described protocol (Farb et al. 2013; Trojsi et al. 2015a, b; 2020).

Images were bias-corrected, tissue-classified, and registered using linear (12-parameter affine) and non-linear transformations (warping) within a unified mode. Subsequently, the warped GM segments were affine-transformed into Montreal Neurological Institute (MNI) space and were scaled by the Jacobian determinants of the deformations. Moreover, modulated images were smoothed with 8-mm full-width half-maximum Gaussian kernel to create the final probability maps. GM atrophy results of comparisons between ALS patients and HCs were familywise error (FWE) corrected at a level of *p* < 0.05. Regarding the VBM analyses, gender effects on regional GM volume were investigated through whole-brain voxel-wise comparisons of the preprocessed GM images of ALS patients and controls by using a full factorial design, which always included total intracranial volume and age as nuisance variable.

## Results

### Demographics and neuropsychological variables

Patients and HC characteristics are reported in Table 1. One-way ANOVAs did not show statistically significant differences in demographic, clinical, and cognitive features in terms of between- and within-sex comparisons (Table 1).

**Table 1:** Descriptive statistics and comparisons between amyotrophic lateral sclerosis and healthy control groups considering the sex

	ALS		HC		*t* test (*p* value)			
	M (*n* = 25)	F (*n* = 20)	M (*n* = 15)	F (*n* = 15)	ALS-M vs. ALS-F	HC-M vs. HC-F	ALS-M vs. HC-M	ALS-F vs. HC-F
Demographics								
Age, years	57.64 (10.16)	57.55 (9.07)	55.47 (8.05)	54.93 (10.40)	0.00 (0.97)	0.02 (0.87)	0.49 (0.48)	0.62 (0.43)
Education, years	9.59 (4.50)	10.28 (4.49)	13.43 (3.04)	12.20 (2.95)	0.23 (0.63)	0.80 (0.37)	4.39 (0.04)	2.01 (0.16)
Clinical features								
Symptom duration, months	10.92 (4.44)	11.60 (3.34)	–	–	0.32 (0.57)	–	–	–
ALSFRS–R total score	39.36 (7.18)	36.55 (5.46)	–	–	2.08 (0.15)	–	–	–
ALSFRS–R subscore								
Bulbar	10.56 (2.69)	10.65 (2.32)	–	–	0.01 (0.90)	–	–	–
Fine motor	8.28 (3.44)	7.10 (2.98)	–	–	1.46 (0.23)	–	–	–
Gross motor	9.36 (2.76)	7.40 (3.51)	–	–	4.38 (0.04)	–	–	–
Respiratory	11.40 (1.82)	11.35 (1.13)	–	–	0.01 (0.91)	–	–	–
Disease progression rate	0.90 (0.83)	1.06 (0.55)	–	–	0.52 (0.47)	–	–	–
UMN score	7.08 (4.41)	8.35 (4.04)	–	–	0.99 (0.32)	–	–	–
Cognitive measures								
MMSE	27.56 (1.76)	28.46 (1.03)	28.60 (1.40)	28.26 (1.03)	2.35 (0.13)	0.54 (0.46)	2.96 (0.09)	0.24 (0.62)
ECAS	104.49 (9.82)	102.71 (10.62)	–	–	0.21 (0.65)	–	–	–

*ALS* amyotrophic lateral sclerosis group, *HC* healthy control group, *M* male, *F* female, *ALSFRS*-R ALS functional rating scale revised, *UMN* upper motor neuron score, *MMSE* mini mental state examination, *ECAS* Edinburgh Cognitive and Behavioural *ALS* screen, significant group difference was reported in **bold** after Bonferroni correction: [for ALS-*M* vs. ALS-*F*: < 0.01 (0.05/12), for HC-*M* vs. HC-*F* ALS-*M* vs. HC-*M* and ALS-*F* vs. HC-*F*: 0.01 (0.05/3)]

### RSN functional connectivity analysis

Across all subjects, the mean FD was below the threshold of 0.5 mm (mean ± st. dev.: 0.090 ± 0.062 mm). In addition, the mean FD was not significantly different between the analyzed experimental groups (two-sample *t* tests: *p* > 0.05). At the selected data dimensionality (40 components per subject), the mean R-index of ICASSO solutions below 1 (mean ± st. dev.: 0.89 ± 0.31) and the mean B-index higher than 99% (mean ± st. dev.: 99.6% ± 0.30%). In addition, neither the mean R- or B-index was significantly different between groups (two-sample *t* tests: *p* > 0.05).

When comparing men to women and ALS patients to HCs, among RSNs, the sensorimotor network (SMN), the default mode network (DMN), the right and left fronto-parietal network (R-, L-FPN) and the salience network (SLN) components showed statistically significant regional between-sex and within-sex (ALS-related) effects in their spatial distribution.

#### SMN

**Fig. 1:**
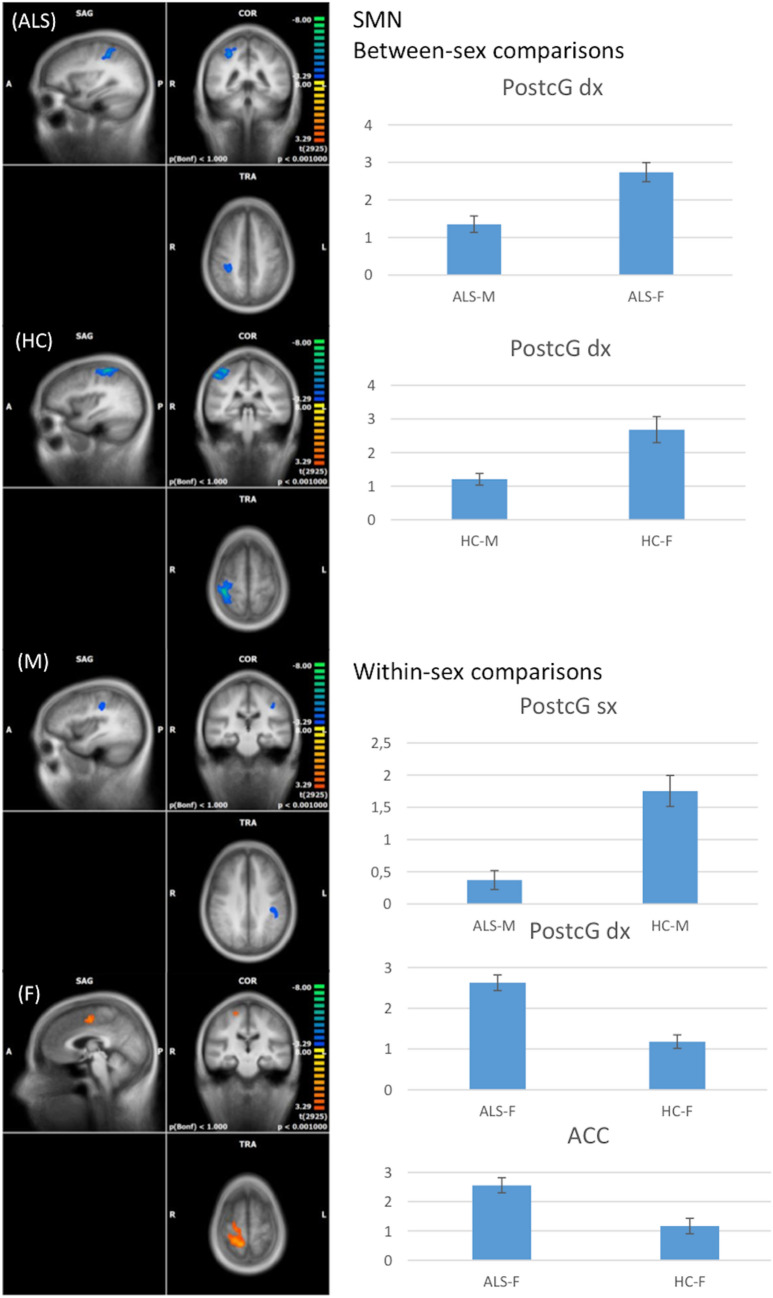
RSNs between-sex (ALS-M vs ALS-F; HC-M vs HC-F) and within-sex (ALS-M vs HC-M; ALS-F vs HC-F) comparisons regarding the sensorimotor network (SMN) (on the left, maps: right-yellow scale for FC increase, blue scale for FC decrease; on the right, bar plots of the average FC levels). *A* anterior; *ACC* anterior cingulate cortex; *clc* cluster-level corrected; *COR* coronal; *F* female; *L* left; *M* male; *P* posterior; *postCg* post-central gyrus; *R* right; *SAG* sagittal; *TRA* transverse

When compared to women with ALS, men with ALS exhibited a decreased functional connectivity (FC) in the right post-central gyrus (cluster-level corrected *p* <  = 0.05, voxel-level *p* <  = 0.001) (Fig. 1).

The same pattern was identified also by between-sex comparison in the HCs group. Within-sex comparison between male ALS patients versus male HCs exhibited a decreased FC in the same area (post-central gyrus) of the left hemisphere (cluster-level corrected *p* <  = 0.05, voxel-level *p* <  = 0.001) (Fig. 1). Conversely, women with ALS showed increased FC in the post-central gyrus and in the anterior cingulate cortex (ACC) in comparison to female HCs (cluster-level corrected *p* <  = 0.05, voxel-level *p* <  = 0.001) (Fig. 1).

#### DMN

**Fig. 2:**
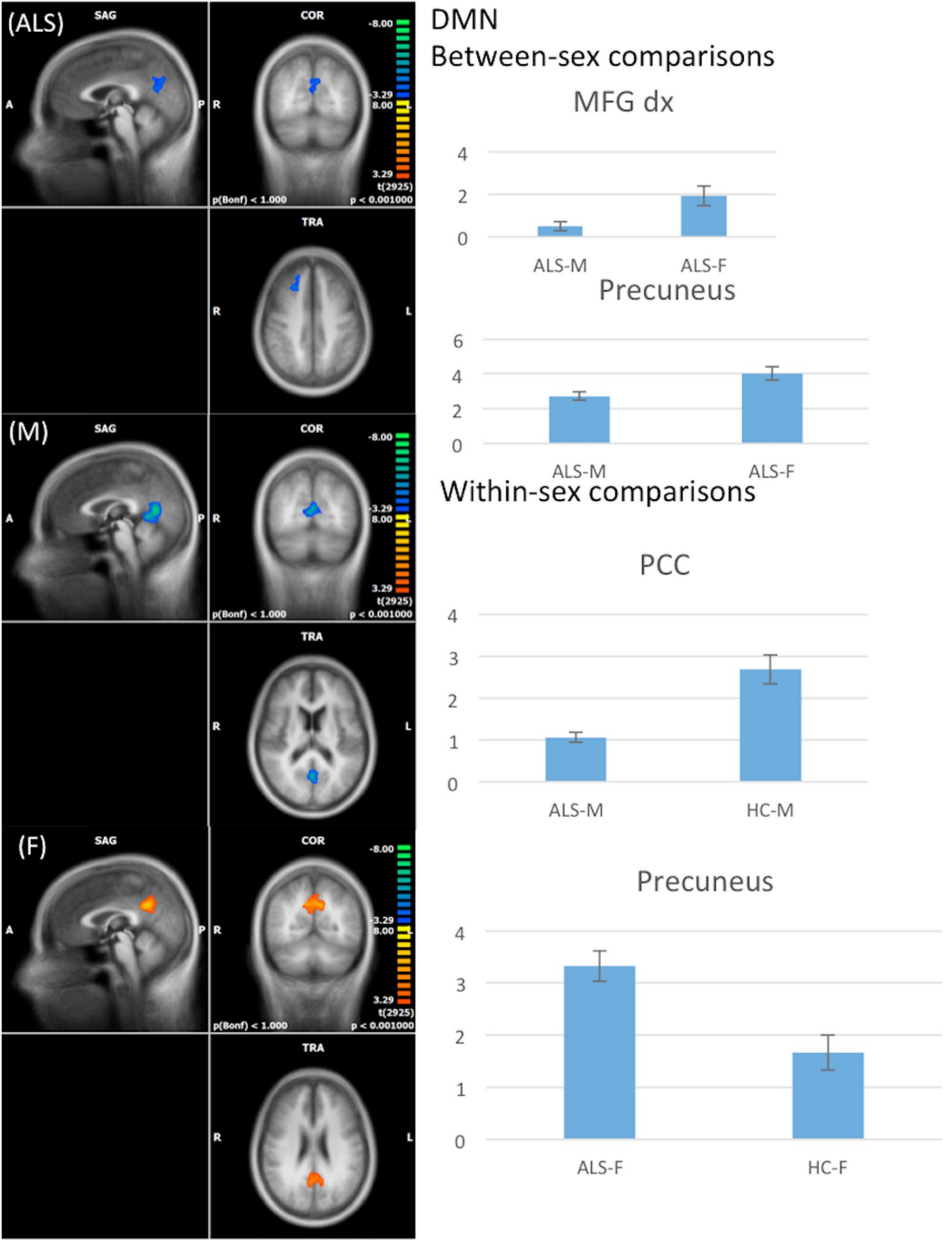
RSNs between-sex (ALS-M vs ALS-F; HC-M vs HC-F) and within-sex (ALS-M vs HC-M; ALS-F vs HC-F) comparisons regarding the default mode network (DMN) (on the left, maps: right-yellow scale for FC increase, blue scale for FC decrease; on the right, bar plots of the average FC levels). *A* anterior; *clc* cluster-level corrected; *COR* coronal; *F* female; *L* left; *M* male; *MFG* middle frontal gyrus; *P* posterior; *PCC* posterior cingulate cortex; *R* right; *SAG* sagittal; *TRA* transverse

When compared to women with ALS, men with ALS exhibited a decreased FC in the right middle frontal gyrus and in the precuneus (cluster-level corrected *p* <  = 0.05, voxel-level *p* <  = 0.001) (Fig. 2), while no significant difference was revealed by comparing female to male HCs.

Within-sex comparisons showed a significant decrease of FC in the posterior cingulate cortex in male patients compared to male HCs and a significant increase of FC in the precuneus in female patients compared to female HCs (cluster-level corrected *p* <  = 0.05, voxel-level *p* <  = 0.001) (Fig. 2).

#### FPNs

**Fig. 3:**
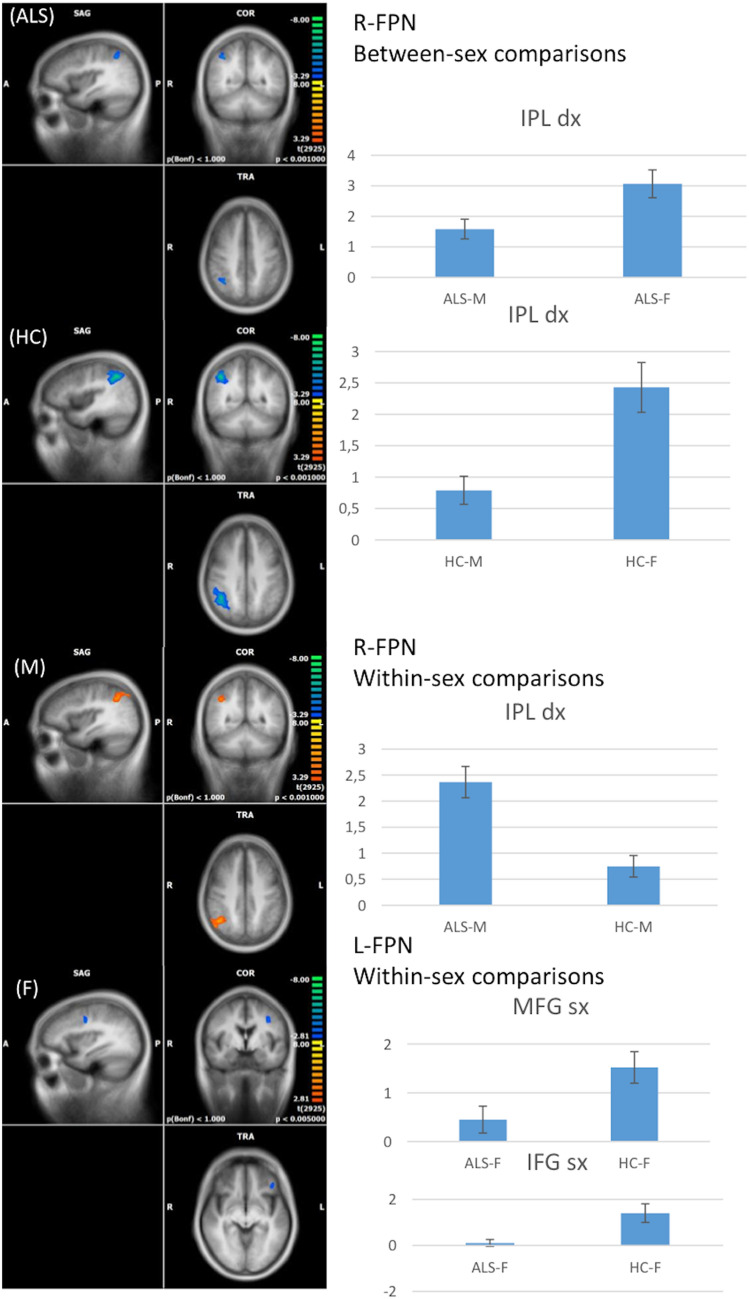
RSNs between-sex (ALS-M vs ALS-F; HC-M vs HC-F) and within-sex (ALS-M vs HC-M; ALS-F vs HC-F) comparisons regarding the right and left fronto-parietal networks (R-, L-FPN) (on the left, maps: right-yellow scale for FC increase, blue scale for FC decrease; on the right, bar plots of the average FC levels). *A* anterior; *clc* cluster-level corrected; *COR* coronal; *F* female; *IFG* inferior frontal gyrus; *IPL* inferior parietal lobule; *L* left; M male; *MFG* middle frontal gyrus; *P* posterior; *R* right; *SAG* sagittal; *TRA* transverse

With regard to the R-FPN, when compared to women with ALS, men exhibited a decreased FC in the right inferior parietal lobule (cluster-level corrected *p* <  = 0.05, voxel-level *p* <  = 0.001) (Fig. 3). The same pattern was identified by comparing male HCs to female HCs.

Within-sex comparisons showed increased FC in the right inferior parietal lobule in male patients compared to male HCs and decreased FC in the right middle frontal gyrus in female patients compared to female HCs (cluster-level corrected *p* <  = 0.05, voxel-level *p* <  = 0.001) (Fig. 3).

With regard to the L-FPN, when compared to women with ALS, men with ALS did not exhibit significant differences in any area of the network, although male HCs, compared to female HCs, showed decreased FC in the left inferior and middle frontal gyrus (cluster-level corrected *p* <  = 0.005). Within-sex comparisons showed decreased FC in the left superior temporal gyrus in the male patients compared to male HCs, and in the inferior and middle frontal gyrus in female patients compared to female HCs (cluster-level corrected *p* <  = 0.05, voxel-level *p* <  = 0.005) (Fig. 3).

#### SLN

**Fig. 4:**
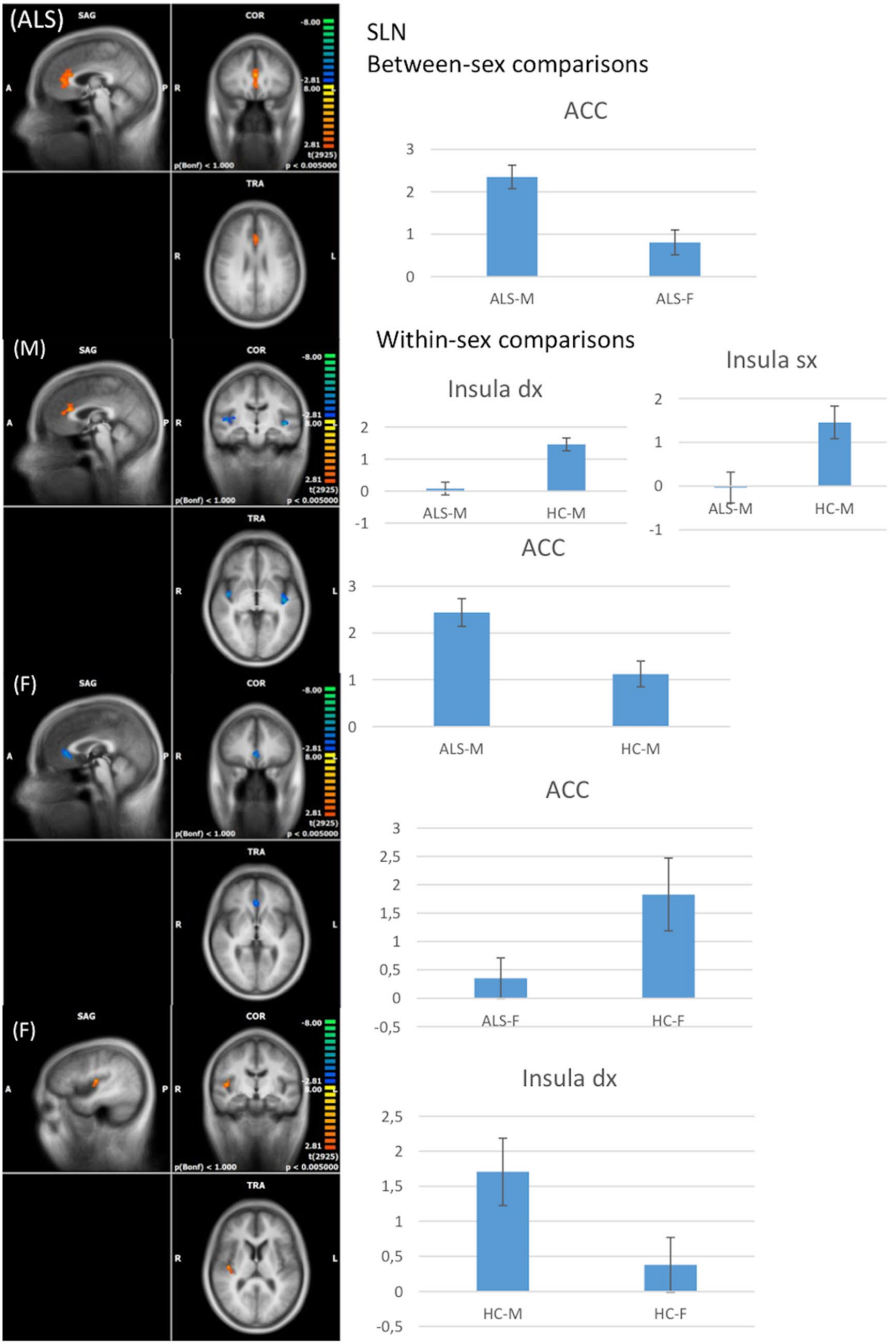
RSNs between-sex (ALS-M vs ALS-F; HC-M vs HC-F) and within-sex (ALS-M vs HC-M; ALS-F vs HC-F) comparisons regarding the salience network (SLN) (on the left, maps: right-yellow scale for FC increase, blue scale for FC decrease; on the right, bar plots of the average FC levels). *A* anterior; *ACC* anterior cingulate cortex; *clc* cluster-level corrected; *COR* coronal; *F* female; *L* left; *M* male; *P* posterior; *R* right; *SAG* sagittal; *TRA* transverse

Between-sex comparisons showed that male patients, compared to female patients, exhibited a decreased FC in the ACC (voxel-level *p* <  = 0.005, cluster-level *p* <  = 0.05) (Fig. 4). Moreover, male HCs showed an increased FC in the right anterior insular cortex in comparison to female HCs (cluster-level *p* <  = 0.05, voxel-level *p* <  = 0.005).

Within-sex comparison revealed a significant increased FC in the right and left anterior insular cortices and decreased FC in the ACC in male patients compared to male HCs (cluster-level *p* <  = 0.05, voxel-level *p* <  = 0.005) (Fig. 4). Finally, female patients showed decreased FC in the ACC in comparison to female HCs (cluster-level *p* <  = 0.05, voxel-level *p* <  = 0.005).

### fALFF analysis

**Fig. 5:**
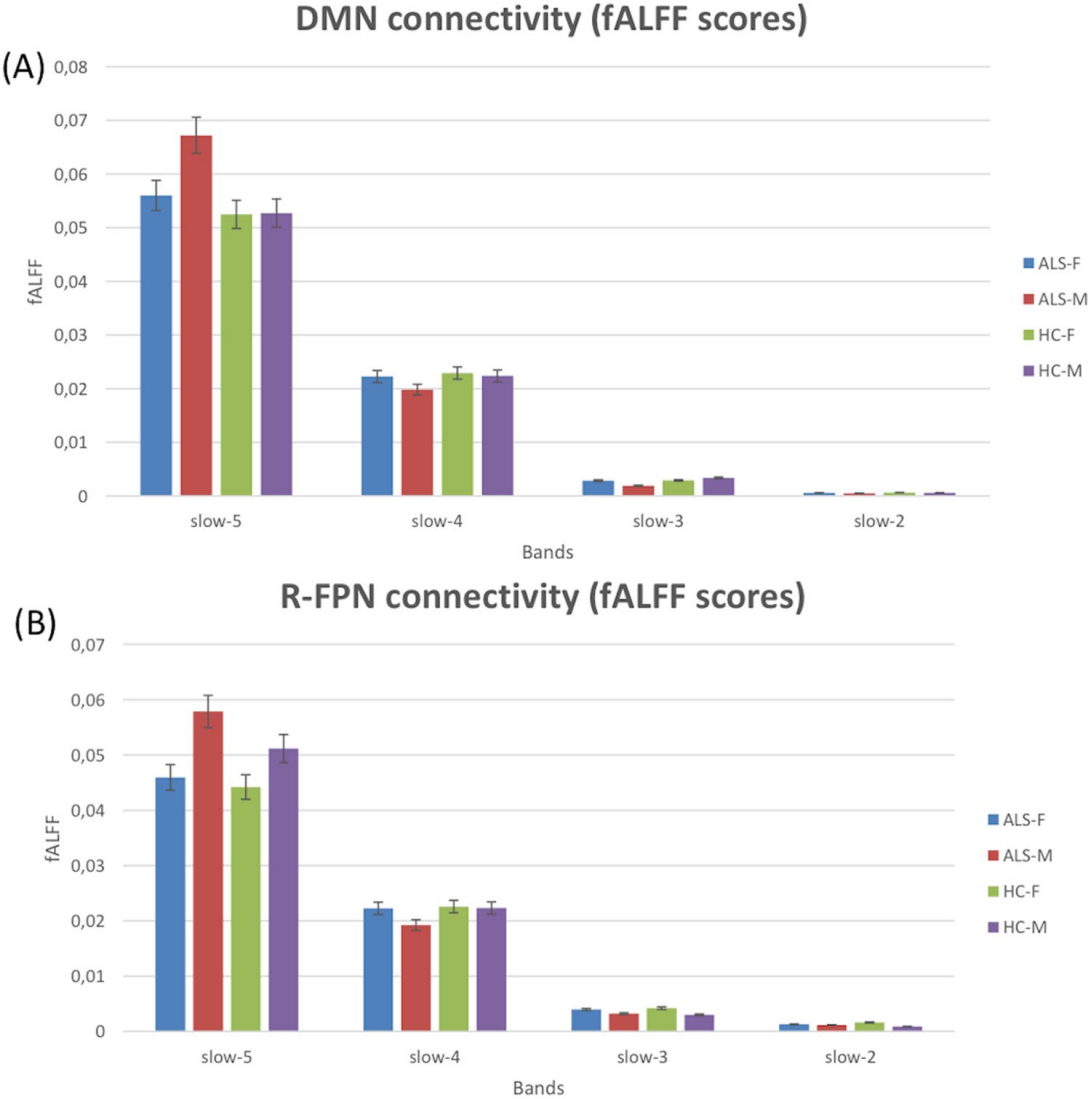
Average spectral power distribution of the DMN (**A**) and R-FPN (**B**) networks time-courses subdivided into four canonical bands in all studied groups (ALS-M, males with ALS; ALS-F, females with ALS; HC-M, male healthy controls; HC-F, female healthy controls) as expressed by the fALFFs

Male ALS patients showed increased fALFF in the DMN in the slow-5 band when compared to both female ALS patients (*p* = 0.04, uncorrected) and male HCs (*p* = 0.02, uncorrected; Fig. 5). Moreover, male ALS patients showed increased fALFF in the R-FPN in the slow-5 band when compared to female ALS patients (*p* = 0.04, uncorrected; Fig. 5). No other fALFF differences were detected between male and female ALS and HCs and between female/male ALS patients and HCs in any spectral band of the other RSNs.

### VBM analysis

**Fig. 6:**
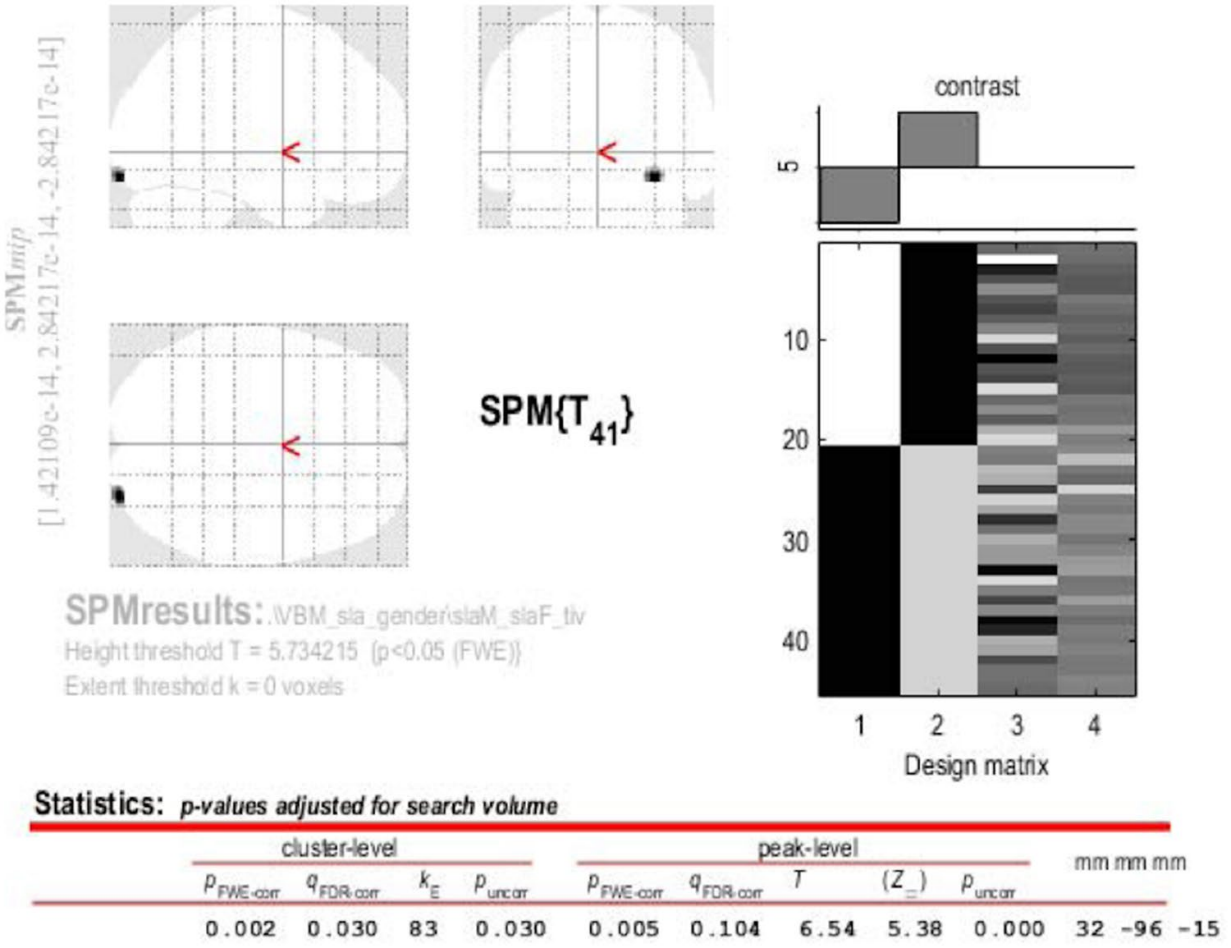
SPM results of between-sex (ALS-M vs ALS-F) VBM analysis: a clusters of GM atrophy is displayed in the right lateral occipital cortex (*p* < 0.05, FWE-corrected)

Between-sex comparisons revealed regional atrophy in right lateral occipital cortex in female ALS patients compared to male ALS patients (*p* < 0.05, FEW-corrected; Fig. 6), and no significant differences when comparing healthy men to healthy women. Finally, within-sex comparisons revealed no significant difference in GM atrophy.

## Discussion

This MRI study investigated, for the first time, the sexual dimorphism of brain RS-fMRI and VBM measures in ALS in comparison to health condition. Our findings, although confirming the evidence of a pattern of widespread brain RS-fMRI abnormalities extended to both motor and extra-motor RSNs, such as SMN, FPNs and SLN, in both female and male patients compared to sex-matched HCs, also revealed reduced FC in the DMN in the right middle frontal gyrus and in the precuneus and GM atrophy in the right lateral occipital cortex selectively in men with ALS compared to women with ALS. Conversely, fALFF analysis revealed an increasing trend of fALFF in the DMN in the slow-5 band in male ALS patients in comparison to both female ALS patients and male HCs.

While structural dimorphism in the human brain is well-described (Delvecchio et al. 2021; Seitz et al. 2021; van Eijk et al. 2020a, b), controversy exists regarding the existence and degree of sex-related differences in brain function. In healthy conditions, several studies have described sex-related differences in fronto-parietal, cingulo-opercular and temporal connections in typically developing adults (Biswal et al. 2010; Zuo et al. 2010b) or individual networks such as the SLN and DMN in healthy aging (Jamadar et al. 2019). A key finding of a recent study by de Lacy et al. (2019), who investigated 650 young adults matched for age and sex to determine the degree of sexual dimorphism in RSNs, was that most intrinsic networks exhibit significant sex-related effects, with both females versus males and males versus females effects usually found within the same network showing larger effects in women, although spreading across greater network territory in men. To note, sexual dimorphism was particularly common in task-positive control networks and in the DMN (de Lacy et al. 2019). In agreement with these findings and others that have found sex-related differences in the DMN in healthy conditions (Biswal et al. 2010; Zuo et al. 2010b), our results revealed that the function of the default mode system may be most strongly influenced by sex in ALS in comparison to other network types. In particular, between-sex comparisons revealed decreased FC in the right middle frontal gyrus and in the precuneus of the DMN only in affected men compared to affected women. Intriguingly, with regard to the posterior component of the DMN, affected men showed decreased FC in the posterior cingulate cortex when compared to unaffected men, while affected women showed an increased FC in the precuneus when compared to unaffected women. Taken together, these findings may corroborate the hypothesis that abnormal modulation of FC in the DMN may be a fingerprint of ALS-related alterations of brain FC, in agreement with previous RS-fMRI findings (Agosta et al. 2013; Trojsi et al. 2015b), additionally outlining that decreased FC in the posterior portion of the DMN is more characteristic of the male sex, recognized as a risk factor for early onset and respiratory and flail limb phenotypes of ALS (Chiò et al. 2020). Moreover, the evidence of adaptive diversity and complementarity of brain FC patterns between the sexes, revealed for the healthy condition (Gur and Gur 2017), seems to be confirmed also in ALS patients. On the other hand, a wide variety of human neurological and neuropsychiatric disorders showing sex-related differences in incidence, prevalence and severity has been associated with between-sex differences in FC modulation within DMN (Mohan et al. 2016).

To note, while ICA of RS-fMRI signals showed decreased FC in the right middle frontal gyrus and in the precuneus of the DMN in affected men compared to affected women, fALFF analysis revealed an increasing trend of fALFF in the DMN in the slow-5 band in male ALS patients compared to both women with ALS and healthy men. These findings represent further evidence that multiple, complementary analytical approaches are valuable for obtaining a more comprehensive characterization of RSNs alterations, from both spatial distribution and spectral composition point of views, as also revealed in previous RS-fMRI analyses of neurodegenerative, psychiatric or painful conditions (De Micco et al. 2019; Wolf et al. 2020; Flodin et al. 2014). In particular, we did not only use the ICA map representing the spatial distribution of the “within-network” functional connectivity, but we also used the ICA time-course representing the common signal fluctuations within this network (i.e., those signal fluctuations that are shared among more active regions). Now, the ICA time-course was further characterized in terms of the fALFF in multiple canonical frequency sub-bands (slow-5, slow-4, slow-3, slow-2). Moreover, by comparing network fALFF between groups across sub-bands, we interrogated the relative contribution of specific sub-bands to the previously mentioned common network time-course and, despite the reduction of the functional connectivity of one specific region to the entire network (e.g., DMN or FPN), the same network (as a whole) showed a reconfiguration of the spectral power of the functional connectivity time-course in terms of a trend toward higher contribution in a specific sub-band (slow-5) in male ALS patients compared to both women with ALS and healthy men. This might be interpreted as a compensatory phenomenon because the reduced contribution from one (spatially localized) region to the network is probably compensated by a relative higher contribution from other (spatially distributed) temporal sources that end up to affect the signal in a lower frequency sub-band (slow-5).

Amplitudes of low frequencies, evaluated by fALFF analysis have been proven to vary according to gender in healthy subjects (Lopez-Larson et al. 2011). Little evidence regarding low-frequency amplitudes have been reported in ALS: Ma et al. (2016) revealed widespread fALFF changes in slow-4 and slow-5 bands and, more recently, Bueno et al. (2019), who performed a whole-brain fALFF and regional homogeneity (ReHo) analyses, described widespread decreased fALFF and ReHo in several motor and sensory regions as well as cingulate, temporal, parietal and occipital areas in ALS patients compared to HCs (Bueno et al. 2019). These previous findings suggested abnormal neural activity in key nodes of SMN, DMN, FPN and SLN. In this regard, our result of an increasing trend of fALFF in DMN and R-FPN in the slow-5 band in male patients compared to female ALS patients recalls previous evidence of increased fALFF in middle and superior frontal gyrus in an ALS cohort compared to HCs, as reported by Ma et al. (Ma et al. 2016). Increased fALFF in several areas of RSNs in ALS patients has been speculatively attributed to activation of compensatory mechanisms in response to the neurodegenerative process (Douaud et al. 2011; Tedeschi et al. 2012; Agosta et al. 2013) or to increased inhibitory function attributed to “interneuronopathy” and reactive astrocytosis (Turner et al. 2012; Do-Ha et al. 2018). Probably, these mechanisms could substantially underlie cortical hyperexcitability and, consequently, increased FC revealed in some areas of brain RSNs in ALS patients compared to HCs (Douaud et al. 2011; Tedeschi et al. 2012; Agosta et al. 2013).

With regard to the brain functional abnormalities distinctive of the male population, decreased FC in the post-central gyri (SMN), in the right inferior parietal lobule (R-FPN) and in the ACC (SLN) were observed in our analysis in both ALS and healthy men compared, respectively, to affected and healthy women. On the other hand, ALS males showed increased FC in the right and left anterior insular cortices and decreased FC in the ACC in the SLN when compared to healthy men. While in our analysis the decreased FC in the ACC in the SLN was observed in both male and female patients compared to sex-matched HCs, in accordance with previous RS-fMRI findings from other cohorts of ALS patients compared to HCs (Trojsi et al. 2015b; Bueno et al. 2019), increased FC in the right and left anterior insular cortices, identified in our analysis only in men with ALS compared to healthy men, has been recently revealed in fast progressing phenotype of ALS in comparison to slow progressing phenotype (Trojsi et al. 2021). Consequently, the increased FC in insular cortices might represent a functional marker of poorer prognosis in male patients. To support and corroborate this hypothesis, in the future, further longitudinal functional and structural MRI analyses in cohorts of genotypically and phenotypically well-characterized men and women with ALS could be performed.

VBM between-sex analysis showed GM atrophy in the right lateral occipital cortex only in men with ALS in comparison to affected women. These results were in accordance with findings from cortical thickness analysis by Bede et al. (2014), who revealed, accounting for diagnosis, a trend of higher age-adjusted cortical thickness in the right parieto-occipital and left mid-frontal regions in females, while males demonstrated higher cortical thickness in the left lingual and left superior temporal regions. Moreover, our findings may explain the inconsistency about occipital lobe involvement in gender-mixed cohorts of ALS: some studies (Kassubek et al. 2005) reported occipital lobe involvement, whereas others (Bede et al. 2016) revealed it as a less affected brain region in ALS. On the other hand, Delvecchio et al. (2021) revealed non overlapping age-related, between-sex GM changes across post-adolescence in multiple cortical areas, including mid-occipital cortices and left inferior temporal gyrus. These results together with our observation of a lateral occipital brain damage in male patients may suggest a sex-related effect on cortical atrophy in ALS recalling that observed in ALS patients carrying *C9orf72* repeat expansions in comparison to shorter disease duration (sporadic) patients (Agosta et al. 2017) and that induced by age in healthy men compared to women (Delvecchio et al. 2021).

On the base of our findings and given the emerging evidence of gender differences in ALS, imaging studies focusing on ALS should invariably include sex as a covariate in their models, even if the groups are matched for gender. Furthermore, in the light of the need of a precision medicine approach, aimed at optimizing and individualizing treatment to the molecular drivers of an individual’s disease and beginning to be considered also in ALS, sex should arise as a variable to be considered in interpretation of data from clinical trials performed in cohorts of ALS patients. In fact, the observed trends of between-sex differences, derived from our and previous MRI analyses, might emerge from this interpretation. Moreover, the interaction between genetic, demographic, environmental, and lifestyle risk factors seems to underlie the pathological process inducing ALS, which would comprise deficits in multiple pathways, reflecting a “multistep” model of disease consistent with a six-step process (Chiò et al. 2018). Among demographic factors, sex has been reported as an independent factor influencing the development of ALS, being higher the risk of developing the disease in men with a trend toward higher frequency in older age (Chiò et al. 2020).

There are limitations to our study. First, the studied sample was relatively small. Second, we were not able to perform a longitudinal MRI study, because of the lacking consent of most patients to repeat the MRI exam. Third, our studied cohort had a mean age comprised between 50 and 60 years and, considering the proposed protective effect of female hormones, a gender study of younger patients might potentially reveal further differences. Moreover, we excluded from our analysis more disabled patients hindered to undergo an MRI exam because of diaphragmatic weakness and therefore we cannot exclude that this might have biased the cohort towards less severely disabled ALS patients. Finally, we did not record continuous blood oxygen saturation via oximetry during MRI scan.

## Conclusions

As neurological research moves toward the ideals of precision medicine, it is of great importance to study sex differences in human brain and to explore how they may influence pathological processes. Our findings might prove original while studying the brain FC effects of the known differences between males and females affected by ALS. Gender-related trends of FC changes are significantly represented in DMN, in terms of reduced FC in the posterior component of the network and increased fALFF in the slow-5 band in men with ALS compared to affected women. Moreover, a right lateral occipital cluster of GM atrophy was reported in men with ALS compared to female ALS patients, recalling GM differences induced by age in healthy men compared to women. Sex was confirmed as an additional dimension of functional and structural heterogeneity in ALS, reflecting the complex neurobiology of ALS in which the sex could play a strong contributing role and outlining the need of accounting for this variable in clinical and neuroimaging studies performed in cohorts of ALS patients.

## Data Availability

All data and materials support the reported claims and comply with standards of data transparency. Data will be made available on reasonable request.
